# Biocompatibility study of different hyaluronan products for intra-articular treatment of knee osteoarthritis

**DOI:** 10.1186/s12891-019-2815-6

**Published:** 2019-09-12

**Authors:** Keiji Yoshioka, Madoka Katayama, Takeo Nishiyama, Kohei Harada, Sawako Takeshita, Yuji Kawamata

**Affiliations:** 1 Central Research Laboratory, SEIKAGAKU CORPORATION, 1253, Tateno 3-chome, Higashiyamato-shi, Tokyo, 207-0021 Japan; 2 CMC Laboratory, Tokyo, Japan; 3 Medical Science Liaison Unit, Seikagaku Corporation, Tokyo, Japan

**Keywords:** Hyaluronan, Avian, Fermentation, Biocompatibility, (1 → 3)-β-D-glucan

## Abstract

**Background:**

Intra-articular (IA) injection of hyaluronic acid (HA) (IA-HA) is a well-recognized treatment option for pain associated with symptomatic knee osteoarthritis (OA). IA-HA products differ in their HA content, molecular weight, cross-linking, and source of HA. These differences are assumed to affect the biocompatibility of the IA-HA products once injected inside the knee joint.

**Methods:**

In the present study, we investigated the biocompatibility of three multiple-injection IA-HA products available in the global market. These included SUPARTZ FX™, a medium range molecular weight HA derived from rooster comb (Avian-HA); ORTHOVISC®, a high range molecular weight HA obtained through biological fermentation (Bio-HA); and SYNVISC®, a high molecular weight cross-linked hyaluronan derived from rooster comb (Avian-CL-HA). Rabbit knee joint tissues were histologically and biochemically examined after IA injection of the products. Furthermore, we compared the amounts of impurities in the IA-HA products.

**Results:**

IA injection of Avian-CL-HA into rabbit knee joints induced the aggregation of inflammatory cells, infiltration of eosinophils, and an increase in the number of cells in the synovial fluid. However, these effects were not seen in the Avian-HA and Bio-HA groups. The residual protein content and the contaminant levels of bacterial endotoxins were below the limit of quantitation in all HA products. Avian-CL-HA contained relatively a large amount of (1 → 3)-β-D-glucan, but this was below the lower limit of quantification in the other HA products.

**Conclusions:**

The present results clearly demonstrate that the biocompatibility of Avian-HA is comparable to that of Bio-HA, and they were both considered to have a favorable safety profile for the treatment of symptomatic OA of the knee. However, immunostimulatory activity was observed after injection of Avian-CL-HA: this might be a result of its unique cross-linking structure and/or the considerable amount of (1 → 3)-β-D-glucan impurity present in the formulation.

## Background

Knee osteoarthritis (OA) is a major common joint disorder that is predicted to become even more prevalent with population ageing [1, 2]. OA is characterized by progressive deterioration of the articular cartilage and a decrease in the rheological behavior of the synovial fluid, which includes a reduction in the molecular weight and concentration of hyaluronic acid (HA), causing decreased elasticity and viscosity of the synovial HA matrix [3, 4]. Intra-articular (IA) injection of HA is a widely used treatment option for pain associated with symptomatic knee OA [5–7]. In the knee joint, HA plays an important role in the lubrication of the joints under both dynamic and static conditions, and in exerting various physiological effects, such as decrease in pro-inflammatory cytokine levels for mitigating cartilage degeneration and reduction of COX-2 production for reducing pain sensation [8–10]. Therefore, it is recognized that IA-HA is a beneficial treatment for OA.

IA-HA products are currently characterized by various properties, including their HA content, molecular weight, cross-linking, and the source of HA. The four major types of HA products are defined by HA source and cross-linking. Our study is focused on three of the multiple-injection products available: SUPARTZ FX™ and SYNVISC® are avian-based products, whereas ORTHOVISC® is produced by bacterial fermentation. In addition, SYNVISC® is cross-linked, whereas SUPARTZ FX™ and ORTHOVISC® are not. SYNVISC® has a unique structure linked via protein and divinyl sulfone (DVS) [11]. All these products are generally well tolerated, and the most common side effects are injection site pain, swelling and/or fluid buildup around the knee. These reactions are typically mild and resolved without treatment.

Rare cases of severe side effects caused by IA-HA are recognized as pseudoseptic reactions. It has been suggested that the unique cross-linked structure of SYNVISC® may be associated with an increased incidence of these reactions [12–14]. The development of pseudoseptic reactions has been reported in patients receiving repeated injections of SYNVISC® [15, 16]; however, it has not been reported that the other avian-derived products induce clear symptom characterized as pseudoseptic reactions so far. On the other hand, Altman et al. reported that patients using biological fermentation-derived HA had fewer cases of acute flare-ups at the injection site than those using avian-derived HA products [17]. These reports have led to confusion as to whether the severe reactions are associated with the source of HA or the existence of a cross-linked structure.

To assist in the understanding of the safety characteristics of IA-HA products, we studied the biocompatibility of three HA products, including Avian-HA (SUPARTZ FX™), Bio-HA (ORTHOVISC®), and Avian-CL-HA (SYNVISC®), in normal rabbit knee joints and compared the amount of impurities in the three products.

## Methods

### Materials

SUPARTZ FX™ was purchased from Seikagaku Corporation (Tokyo, Japan). ORTHOVISC® was from Anika Therapeutics, Inc. (Bedford, MA). SYNVISC® was from Sanofi K.K. (Tokyo, Japan). Details of these products are given in Table 1. Water for Injection (WFI) and saline (Otsuka Normal Saline) was from Otsuka Pharmaceutical Factory, Inc. (Tokushima, Japan). The Glucatell® and Pyrochrome® plus Glucashield® kits were from Associates of Cape Cod, Inc. (Falmouth, MA), and used for (1 → 3)-β-D-glucan and endotoxin detection, respectively. The DC (detergent compatible) protein assay kit II was from Bio-Rad Laboratories, Inc. (Hercules, CA), and was used for protein detection in each HA product. The Pierce™ BCA Protein Assay kit was from Thermo Fisher Scientific (Waltham, MA), and was used for protein detection in synovial fluid in the biocompatibility study. Endo (1 → 3)-β-D-glucanase was prepared according to the method of Tanaka et al. [18].

**Table 1:** HA products used in this study

Brand name (Lot number)	Type	Concentration	Origin	Molecular weight^a^
SUPARTZ FX™ (4X7Z01)	Natural HA	10 mg/mL (2.5 mL)	Avian	620–1170 kDa
ORTHOVISC® (N170038B)	Natural HA	15 mg/mL (2 mL)	Fermentation	1000–2900 kDa
SYNVISC® (6RSP017)	Cross-linked HA (protein and DVS)^b^	8 mg/mL (2 mL)	Avian	hylan A; 6000 kDa hylan B; > 6000 kDa

^a^Molecular weight supplied by the manufacturer

^b^Hylan A has protein-mediated cross-linking, hylan B is composed of hylan A with additional DVS-mediated cross-linking

### Animals

Male New Zealand White rabbits (12 weeks old) were purchased from Oriental Yeast Co., Ltd. (Tokyo, Japan). The animals were housed under specific pathogen-free conditions at a room temperature between 20 °C and 26 °C, and humidity of approximately 50% on a 12-h/12-h light/dark cycle. The animals were quarantined and acclimatized for 1 week before dosing. The animal studies were reviewed and approved by the Institutional Animal Care and Use Committee of Seikagaku Corporation (approval number, 72–306) and performed under the animal husbandry/management system in an appropriate environment with animal protection/welfare in mind.

### Biocompatibility study in normal rabbit knee joints

A biocompatibility study was conducted in accordance with our previous study [19]. Twenty-one rabbits were divided into four groups (*n* = 6 or 3) based on body weight and anesthetized with an intravenous injection of mixed anesthesia containing midazolam (Dormicum, Astellas Pharma Inc., Tokyo, Japan), xylazine (Selactar 2%, Bayer Yakuhin Ltd., Osaka, Japan), butorphanol (Vetorphale, Meiji Seika Pharma Co., Ltd., Tokyo, Japan), and saline (1:2:1:2), in a volume of 2 mL/animal. The animals received three consecutive weekly intra-articular injections of 0.25 mL of either saline, Avian-HA, Bio-HA, or Avian-CL-HA into both knee joints. All the rabbits were euthanized by exsanguination under deep anesthesia induced with the above-mentioned mixed anesthesia intravenous injection at 15 days after the first injection. Both knee joints were collected. Synovial lavage fluid was collected from the right knee joint by washing the joint cavity three times with 0.5 mL washing buffer (1 mM ethylenediaminetetraacetic acid in phosphate-buffered saline). The total number of cells in the synovial fluid was counted using a light microscope. For characterization of the cell types (monocytes, lymphocytes, and heterophils), the synovial fluid was smeared on a slide, stained with Diff-Quick (Sysmex Corporation, Hyogo, Japan), and then observed with a light microscope. The total protein content in synovial fluid was measured using the Pierce™ BCA Protein Assay kit. The synovium was collected from the left knee joint and was fixed in 10% neutral buffered formalin. Fixed samples were processed, embedded in paraffin, sectioned, and stained with hematoxylin and eosin (H&E) for histological examination. Blinded histological scoring was performed independently by two investigators. Inflammatory reaction with eosinophil infiltration was scored on a 4-point scale, from Grade 0 (no change) to Grade 3 (severe change). Synovial thickening was scored using criteria given in a previous report [19] (Table 2). The numbers of evaluated samples were *n* = 6 per each group except for the saline group, which was *n* = 3.

**Table 2:** Histological scoring criteria

	Histological findings	Score
Synovial thickening	Lining cell layer 1–2 cells thick	0
	Lining cell layer 3–5 cells thick	1
	Lining cell layer 6–8 cells thick and/ or mild increase in cellularity	2
	Lining cell layer > 9 cells thick and/ or severe increase in cellularity	3
Inflammatory cell infiltration	None	0
	Slight	1
	Moderate	2
	Severe	3

### Determination of protein levels

The protein level in each HA product was determined by the DC protein assay kit II according to the manufacturer’s instructions. Each HA product was diluted two-fold with purified water and incubated with the attached assay reagent for a minimum of 15 min at room temperature. The absorbance of the reaction mixture was measured in triplicate at 750 nm with a spectrophotometer (UV-1700; Shimadzu Corporation, Kyoto, Japan). The protein concentration range for the standard curve was 5–125 μg/mL. The sensitivity of the assay is defined as the lowest concentration used in the standard curve. To determine protein recovery, each HA product was spiked with a known amount of bovine serum albumin (BSA) and assayed, and the recovery rate was calculated.

### Bacterial endotoxin test

Bacterial endotoxin levels were determined using the kinetic chromogenic *Limulus Amoebocyte Lysate* (LAL) assay (Pyrochrome® reconstituted with Glucashield® buffer). Avian-HA was diluted twice with Water for Injection. Bio-HA and Avian-CL-HA were diluted 10 times in the same manner. Briefly, 50 μL of test substance was transferred to each well of a 96-well microtiter plate, and the lysate test solution was added at 50 μL/well. The optical density was read at 405 nm in a plate reader (Wellreader MP-96; Seikagaku Corp., Tokyo, Japan). The endotoxin concentration range for the standard curve was 0.005–0.5 EU/mL. The sensitivity of the assay is defined as the lowest concentration used in the standard curve. To determine endotoxin recovery, each HA product was spiked with a known amount of endotoxin and assayed, and the recovery rate was calculated.

### Determination of (1 → 3)-β-D-glucan levels

(1 → 3)-β-D-glucan levels were determined using the Glucatell® kit according to manufacturer’s instructions. Avian-HA, Bio-HA and Avian-CL-HA were diluted 10-, 30-, and 300-fold, respectively, with water certified free of interfering glucan. The Glucatell/sample mixture was incubated at 37 °C ± 1 °C in a microplate reader (ELx808IU, BioTek Instruments, Inc., Winooski, VT) equipped with a 405 nm filter and analyzed over time. The (1 → 3)-β-D-glucan concentration range for the standard curve was 3.125–50 pg/mL. The limit of quantitation for (1 → 3)-β-D-glucan was 1.8 pg/mL (lower limit of quantitation) and 50 pg/mL (upper limit of quantitation). To determine (1 → 3)-β-D-glucan recovery, each HA product was spiked with a known amount of (1 → 3)-β-D-glucan and assayed, and recovery rate was calculated. The kit contains highly purified (1 → 3)-β-D-glucan derived from pachyman (from *Poria cocos*) as a standard substance.

### Statistical analyses

All statistical analyses were performed using the Statistical Analysis System (SAS Institute Inc., Cary, NC). Parametric Tukey’s test was used for the protein contents and cell numbers in synovial lavage fluid and histological scores. The significance level was set at 5% (two-tailed). Data were represented as the mean and standard error of the mean (SE). For spike and recovery analysis, recovery rate (%) was calculated by dividing the measured value by the spiked value after subtracting any baseline signal from the measured sample.

## Results

### Protein content of synovial fluid

**Fig. 1:**
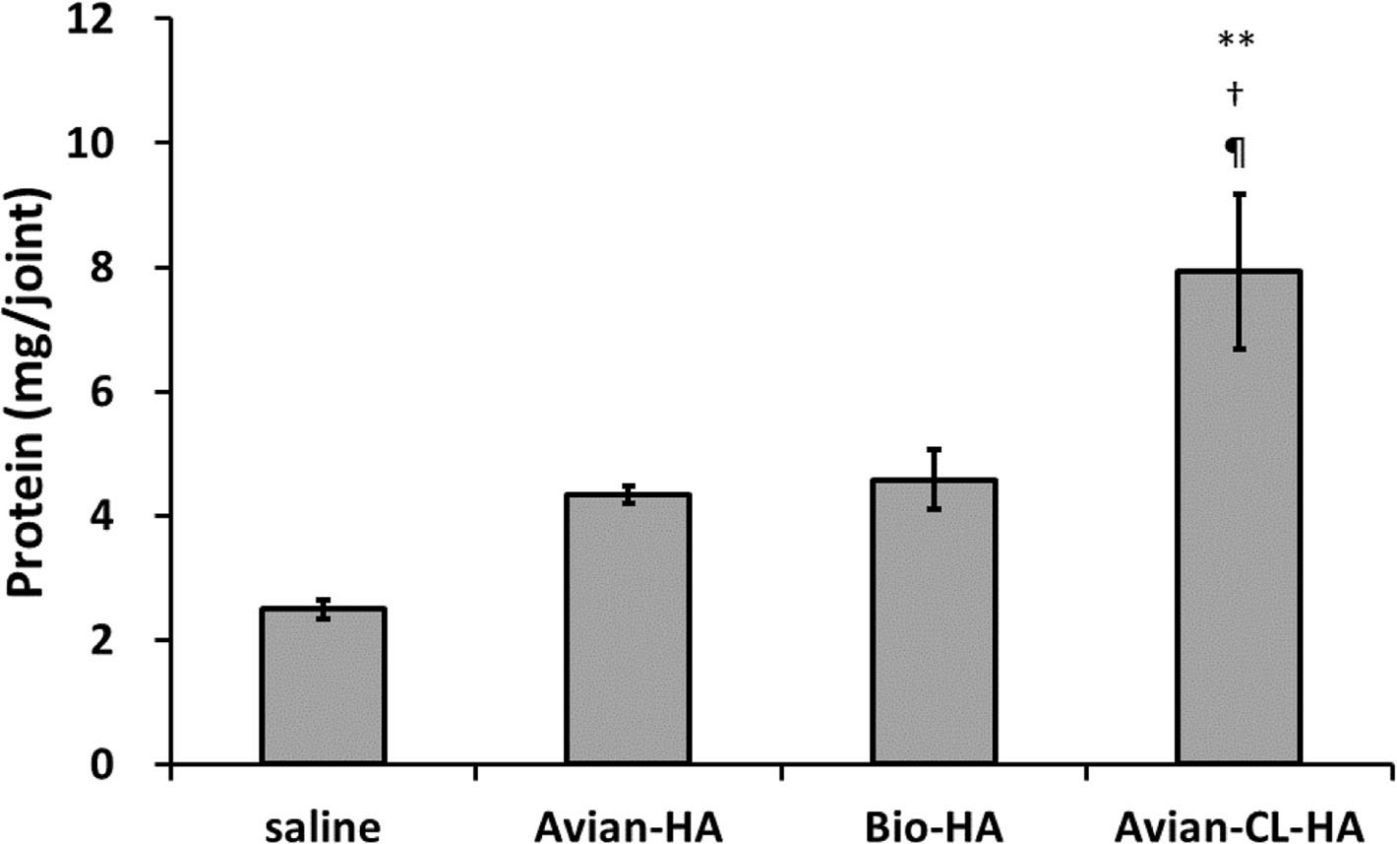
Protein content of synovial fluid in rabbits after three weekly injections of the HA products. Values represent the mean ± SE (*n* = 6; except for the saline group, *n* = 3). **P < 0.01 vs. saline, †*P* < 0.05 vs. Avian-HA, ¶*P* < 0.05 vs. Bio-HA

The mean protein content of synovial fluid was 2.5 ± 0.2 mg/joint in the saline group. In the Avian-HA and Bio-HA groups it was higher, at 4.3 ± 0.1 and 4.6 ± 0.5 mg/joint, respectively, although the increase was not significant (Fig. 1). By contrast, the protein content in the Avian-CL-HA group was 7.9 ± 1.2 mg/joint, significantly higher than that in the saline, Avian-HA, and Bio-HA groups (parametric Tukey’s test, **P < 0.01 [vs. saline], †*P* = 0.01 [vs. Avian-HA], ¶*P* = 0.02 [vs. Bio-HA]).

### Synovial fluid cell counts

**Fig. 2:**
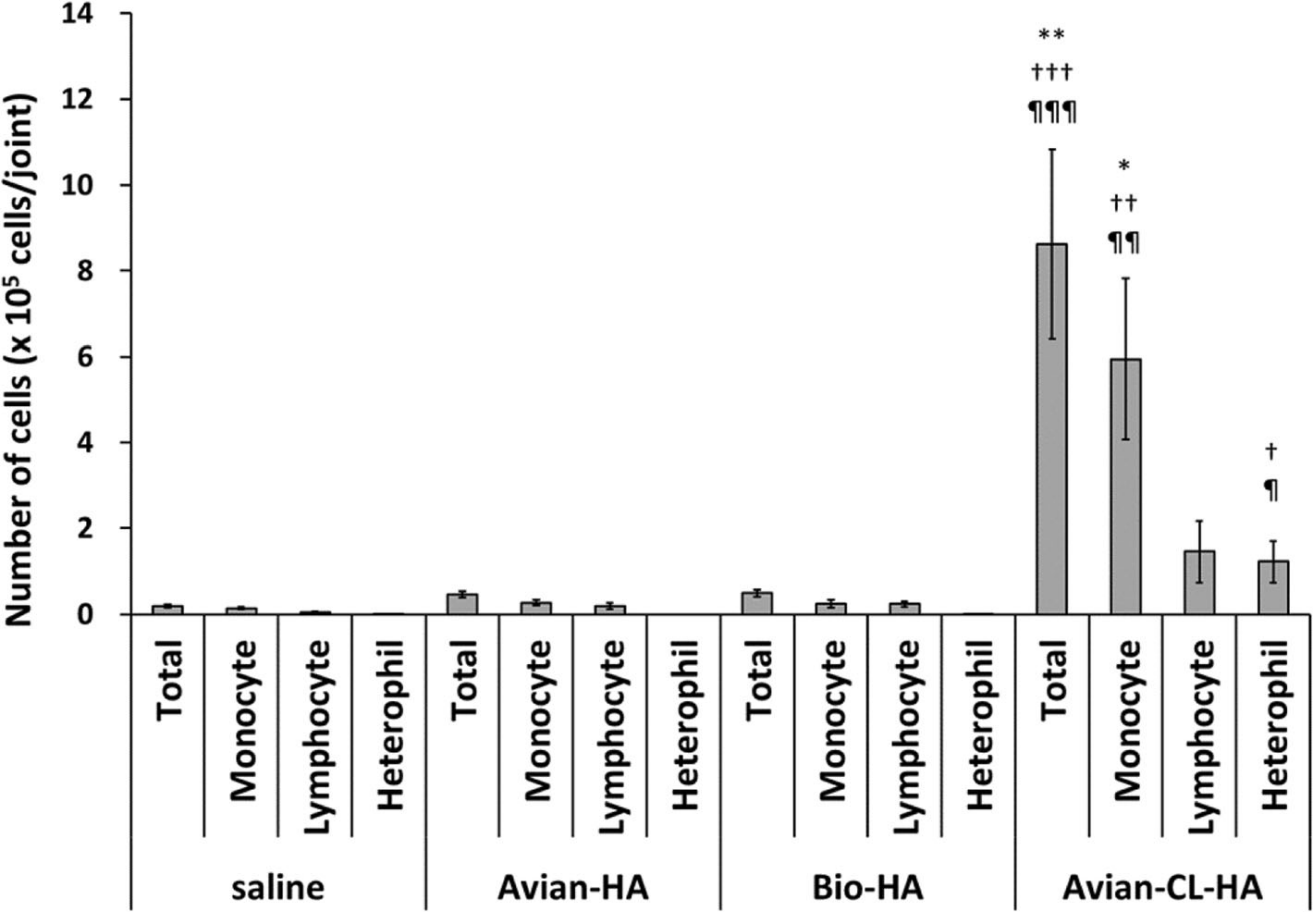
Cell recruitment in synovial fluid in rabbits after three weekly injections of the HA products. Values represent the mean ± SE (*n* = 6; except for the saline group, *n* = 3). **P < 0.01, *P < 0.05 vs. saline; †††*P* < 0.001, ††*P* < 0.01, †*P* < 0.05 vs. Avian-HA; ¶¶¶*P* < 0.001, ¶¶*P* < 0.01, ¶*P* < 0.05 vs. Bio-HA

In the Avian-HA and Bio-HA groups, three consecutive weekly injections did not induce a significant increase in any cell type in the synovial fluid (Fig. 2). In the Avian-CL-HA group, there was a significant increase in the average number of total cells (8.63 × 10^5^), monocytes (5.95 × 10^5^), and heterophils (1.22 × 10^5^), compared to the saline group.

### Tissue responses of normal rabbit knee joints to HA products

**Fig. 3:**
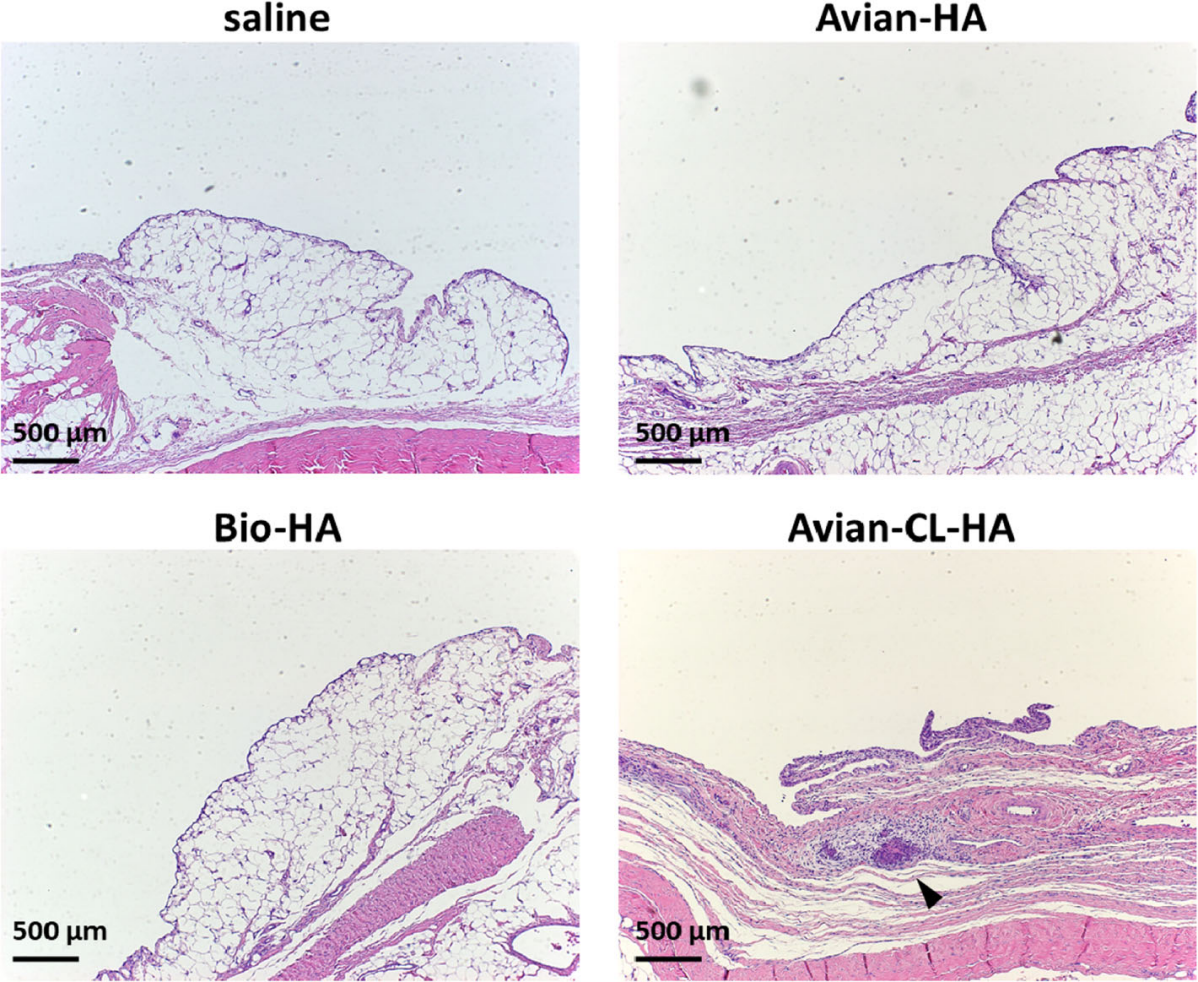
Histological appearance of rabbit knee joints after three weekly injections of the HA products. Sections are stained with H&E and shown at 10× magnification. The arrowhead indicates the aggregation of inflammatory cells. The scale bar indicates 500 μm

**Fig. 4:**
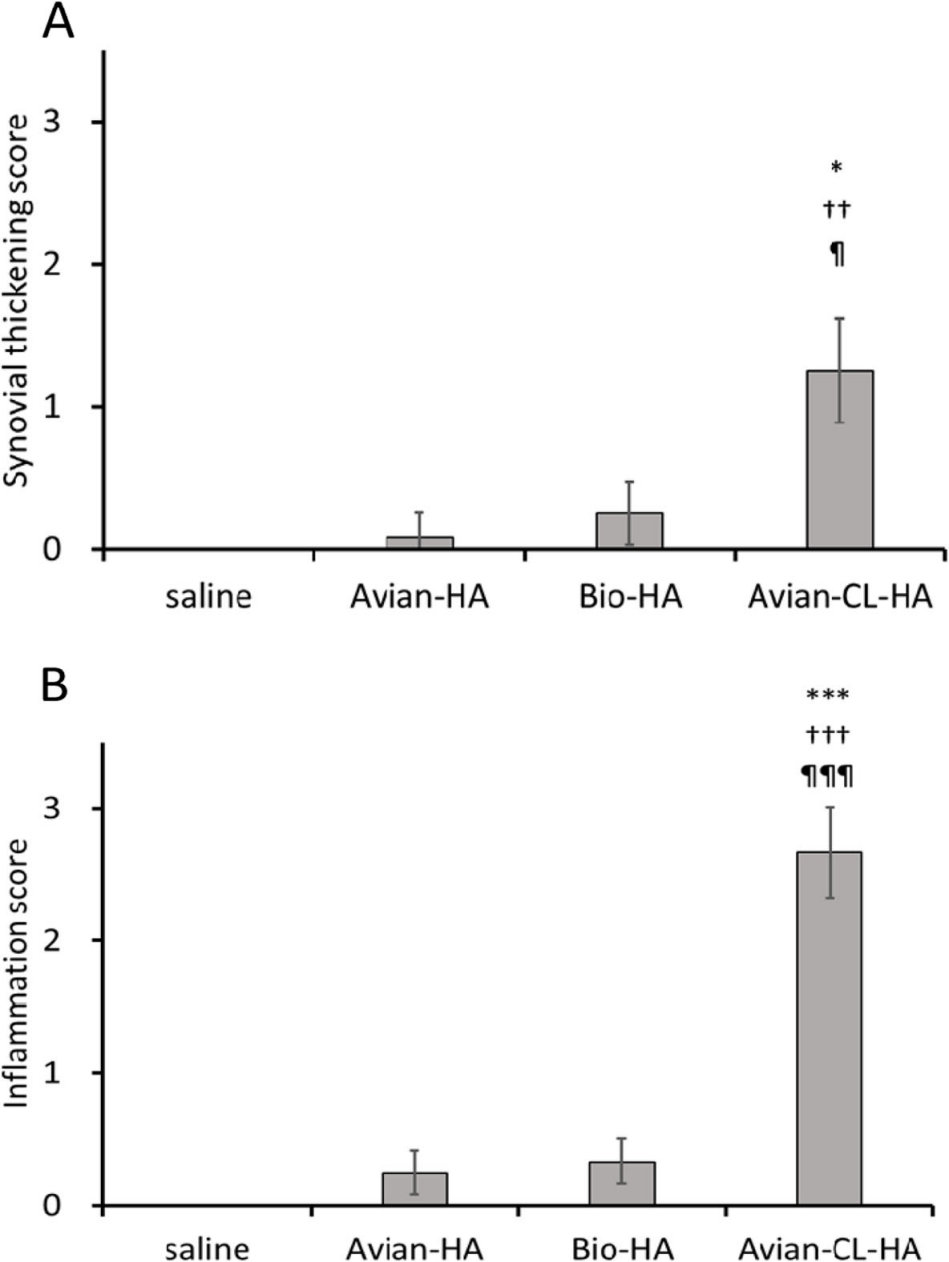
Histological investigation of rabbit knee joints after three weekly injections of the HA products. Values represent the mean ± SE (*n* = 6; except for the saline group, *n* = 3). **a** The average synovial thickening score was increased in the Avian-CL-HA group and was significantly higher than those of the saline, Avian-HA, and Bio-HA groups. **b** The inflammation score was increased in the Avian-CL-HA group and was significantly higher than for the other groups. ***P < 0.001, *P < 0.05 vs. saline; †††*P* < 0.001, ††*P* < 0.01 vs. Avian-HA; ¶¶¶*P* < 0.001, ¶*P* < 0.05 vs. Bio-HA

The histological appearance of the synovium is shown in Fig. 3. One day after three weekly injections of Avian-HA and Bio-HA, no histological changes were detectable in the synovium of treated animals when compared with that of the saline group. A slight proliferation of the synovial cells was seen after injections of Avian-HA and Bio-HA as well as saline; however, eosinophil infiltration was not observed. After three injections of Avian-CL-HA, marked stratification of synovial lining cells, prominent cellular infiltration including eosinophils, and edema in the synovial tissues were observed in all animals. Furthermore, aggregations of inflammatory cells were observed in three of the six animals treated with Avian-CL-HA (black arrowhead). Histological scores are shown in Fig. 4.

### Protein content of HA products

To test whether our assays could efficiently recover protein from the HA products, we performed spike and recovery experiments in which a known amount of bovine serum albumin (BSA) was added to each HA product, and the protein content was determined thereafter. The recovery was close to 100%, ranging from 102 to 109%. As shown in Table 3, Avian-HA, Bio-HA, and Avian-CL-HA all had protein levels that were below the lower limit of quantification (< 10 μg/mL).

**Table 3:** Concentrations of protein, endotoxin, and (1 → 3)-β-D-glucan in the HA products

Product	Protein	Endotoxin	(1 → 3)-β-D-glucan
SUPARTZ FX™ (Avian-HA)	< 10 μg/mL	< 0.01 EU/mL	< 0.018 ng/mL
ORTHOVISC® (Bio-HA)	< 10 μg/mL	< 0.05 EU/mL	< 0.054 ng/mL
SYNVISC® (Avian-CL-HA)	< 10 μg/mL	< 0.05 EU/mL	3.4 ng/mL

### Bacterial endotoxin test

Spike and recovery experiments were performed to determine the accuracy of the method. Recovery rate varied between 76% (Bio-HA) and 151% (Avian-CL-HA). As shown in Table 3, all the HA products had endotoxin levels that were below the lower limit of quantification (< 0.05 EU/mL; except for Avian-HA, < 0.01 EU/mL).

### (1 → 3)-β-D-glucan content of HA products

Spike and recovery experiments were performed to determine the accuracy of the method. Recovery rates varied between 81% (Avian-HA) and 111% (Avian-CL-HA). As shown in Table 3, Avian-HA and Bio-HA had (1 → 3)-β-D-glucan levels that were below the lower limit of quantification (< 0.018 ng/mL and < 0.054 ng/mL, respectively). However, Avian-CL-HA contained 3.4 ng/mL (1 → 3)-β-D-glucan. To confirm the specificity of the test, Avian-CL-HA (100 μL) diluted 100-fold with water certified free of interfering glucan was mixed with 5 μL of 1.17 U/mL endo-(1 → 3)-β-D-glucanase and incubated at 37 °C for 1 h. As a result, 94.0% of the glucan signal was lost, confirming that the measured value for Avian-CL-HA was derived from (1 → 3)-β-D-glucan.

## Discussion

In the present study, we selected three multiple-injection IA-HA products for evaluation which are highly prescribed globally. SYNVISC®, which has a unique safety concern as it causes rare pseudoseptic reactions, is a cross-linked HA derived from rooster comb (Avian-CL-HA). SUPARTZ FX™ and ORTHOVISC® were selected as representative products containing natural HA, derived from rooster comb (Avian-HA) and biological fermentation (Bio-HA), respectively.

In the knee joints of rabbits, Avian-HA and Bio-HA showed favorable biocompatibility compared with Avian-CL-HA. In the Avian-HA and Bio-HA groups, no increase in protein levels was observed in synovial fluid after administration of the products. As for the cell counts in synovial fluid, the patterns of cell recruitment in Avian-HA- and Bio-HA-treated animals were similar to those of the saline group. In the histological examinations, proliferative changes in the synovial surface layer were observed in both groups, but no significant changes were observed as compared with those in the saline-treated group, indicating that they were physiological reactions to the injection stimuli. These results suggest that biocompatibility of Avian-HA is comparable to Bio-HA. However, intra-articularly administered Avian-CL-HA induced severe inflammatory reactions accompanied by monocyte and heterophil infiltration and a significant increase in the number of cells in the synovial fluid. Histologically, aggregation of inflammatory cells, including eosinophils, was observed in the synovium of three animals out of six.

In a previous study, Schiavinato et al. assessed the biocompatibility of the avian-derived HA products, HYALGAN® and ARTZ® (also known as SUPARTZ FX™), in a rabbit model similar to that used in this study. They demonstrated that neither of these HA products increased the number of infiltrating inflammatory cells in synovial fluid [20]. Our previous study showed that intra-articular injection of hylan G-F 20 (SYNVISC®) into rabbit knee joints induced granulomatous inflammation, eosinophil infiltration, and a significant increase in the number of cells in the synovial fluid [19]. The present findings were similar to those previous results. Taken together, these studies show that SYNVISC® has a unique safety concern associated with immunostimulation, whereas there is no difference between Avian-HA and Bio-HA biocompatibility. Therefore, the pseudoseptic reactions reported in case reports on SYNVISC® might not be caused by the source of HA.

SYNVISC® is composed of 80% (v/v) hylan fluid (hylan A) and 20% (v/v) hylan gel (hylan B). SYNVISC® has a unique cross-linked structure in which hylan A has protein-mediated cross-linking and hylan B has additional DVS-mediated cross-linking. The histological examinations in several case reports on SYNVISC® show the development of granulomatous inflammation encompassing the hylan polymer in the knee joint, a typical histopathological observation for pseudoseptic reactions. It has been suggested that the low biodegradability of hylan polymer may be associated with the development of granulomatous inflammation [13, 14, 21]. In addition, our previous animal study revealed that another Avian-CL-HA product, GEL-ONE®, which has a cross-linking structure via cinnamic acid, showed more favorable biocompatibility and less immunogenicity compared to SYNVISC® [19]. These results support an association between the unique cross-linking structure and the unique safety concern for SYNVISC®.

Another factor related to safety is the presence of impurities in IA-HA products. In the present study, the levels of protein were below the lower limit of quantification in all the tested HA products (< 10 μg/mL). Regarding residual endotoxin levels, the previous study reported that SYNVISC® and ARTZ® contained 0.19915 ± 0.0741 and 0.0045 ± 0.0001 EU/mL of endotoxins, respectively [22]. Our study showed that the endotoxin levels in all products were below the lower limit of quantification (0.01–0.05 EU/mL: the differences in the lower limit of quantification were due to the differences in the test sample dilutions). We speculate that the discrepancies between the two studies may result from a difference in the endotoxin quantitation methods used and/or a decrease in the amount of impurities in the product. In the previous study [22], the Toxicolor™ system LS-50 M kit (Seikagaku Corporation) was used for measuring endotoxin in HA products. However, the Toxicolor™ system LS-50 M kit contains a serine protease zymogen factor G, which directly reacts with (1 → 3)-β-D-glucan, in addition to a lipopolysaccharide (LPS)-sensitive serine protease zymogen factor C [23]. Therefore, if (1 → 3)-β-D-glucan is present in the sample, the kit will detect it, giving a false-positive reading for endotoxins. In this study, we used Pyrochrome® plus Glucashield® buffer kit in which the specificity for endotoxin is greatly improved. It is reactive only to endotoxin, not to (1 → 3)-β-D-glucan [24]. Thus, Ohshima et al. may have detected the presence of (1 → 3)-β-D-glucan, rather than endotoxin contamination, in SYNVISC®.

(1 → 3)-β-D-glucan is a polysaccharide composed of D-glucose monomers linked by (1 → 3) beta-glycosidic bonds. It is a characteristic fungal cell wall constituent. It has been reported that fungi-derived (1 → 3)-β-D-glucan modulates various aspects of immunity [25–28]. Aside from fungi, dialysis membranes and filters made from cellulose are occasionally reported to contain (1 → 3)-β-D-glucan that may be solubilized during use. Therefore, the use of cellulose membranes during the manufacturing process is reported to result in significant increases of (1 → 3)-β-D-glucan in pharmaceutical products [29, 30]. In fact, the use of specific immunoglobulin products [31], cotton gauze, and sponges in surgeries [32] are reported to increase serum (1 → 3)-β-D-glucan levels. This is in addition to some drugs having a (1 → 3)-β-D-glucan formulation (lentinan, crestin, scleroglucan, and schizophyllan). Health risks associated with (1 → 3)-β-D-glucan contamination in pharmaceutical products were discussed at the Parenteral Drug Association annual meeting in 2017. Currently, endotoxin levels are strongly regulated and strictly tested for pharmaceutical products (ICH Q6A and Q6B Specifications, 1999 [33, 34]). However, little is known about the clinical significance of (1 → 3)-β-D-glucan contamination, and considerable uncertainty exists over the level at which immunostimulation may occur. Thus, there are no guidelines available on acceptable levels of (1 → 3)-β-D-glucan. In our study, only Avian-CL-HA, containing (1 → 3)-β-D-glucan, induced an increase in inflammatory cells and protein in synovial fluid, and the aggregation of inflammatory cells in the synovium. Although the possibility still remains that the specific structure of the cross-linking causes the inflammatory reactions, the present results suggest another possibility, which is that (1 → 3)-β-D-glucan in intra-articularly injectable preparations may trigger an inflammatory response. It may be safer to monitor the concentration of (1 → 3)-β-D-glucan included in IA-HA products.

The present study clearly demonstrates that Avian-CL-HA contains a high level of (1 → 3)-β-D-glucan contamination and suggests a possible link between this and the inflammatory response. Although the mechanism of pseudoseptic reactions following repeated SYNVISC® injections has not yet been elucidated, the reactions may not be caused by an avian-derived protein, but instead may be due to (1 → 3)-β-D-glucan content, the cross-linking structure in Avian-CL-HA, or both of these factors.

The limitation of this study was that we could not directly refer to the treatment of symptomatic knee OA because the findings were derived from an animal study. Additionally, it was not clear what caused the inflammatory response after Avian-CL-HA administration. Future research should focus on assessing the biocompatibility of extracted (1 → 3)-β-D-glucan from Avian-CL-HA. Furthermore, analyzing the correlation between the protein-mediated cross-linking structure and its biocompatibility may be useful in clarifying the mechanism of the immunostimulatory activity of Avian-CL-HA. These findings will provide new insights into the safety characteristics of IA-HA products.

## Conclusions

The present results clearly demonstrate that the biocompatibility of Avian-HA is comparable to that of Bio-HA. However, immunostimulatory activity was observed after injection of Avian-CL-HA: this might be a result of its unique cross-linking structure and/or the considerable amount of (1 → 3)-β-D-glucan impurity present in the formulation.

## Data Availability

The dataset supporting the conclusions of this article is stored in Seikagaku Corporation, Tokyo, Japan. Further inquiries on the data may be submitted to Keiji Yoshioka (keiji.yoshioka@seikagaku.co.jp).

## Abbreviations

Avian-CL-HA: SYNVISC®, a high molecular weight cross-linked hyaluronan derived from rooster comb
Avian-HA: SUPARTZ FX™, a medium range molecular weight HA derived from rooster comb
Bio-HA: ORTHOVISC®, a high range molecular weight HA obtained through biological fermentation
H&E: Hematoxylin and eosin
HA: Hyaluronic acid
IA: Intra-articular
OA: Osteoarthritis
